# Fabrication and Manipulation of Non-Spherical Particles in Microfluidic Channels: A Review

**DOI:** 10.3390/mi13101659

**Published:** 2022-10-02

**Authors:** Di Jiang, Shaowei Liu, Wenlai Tang

**Affiliations:** 1 College of Mechanical and Electronic Engineering, Nanjing Forestry University, Nanjing 210037, China; 2 Jiangsu Yuyue Medical Equipment and Supply Co., Ltd., Danyang 212300, China; 3 School of Electrical and Automation Engineering, Jiangsu Key Laboratory of 3D Printing Equipment and Manufacturing, Nanjing Normal University, Nanjing 210023, China

**Keywords:** microfluidics, non-spherical particle, particle manipulation

## Abstract

Non-spherical shape is a general appearance feature for bioparticles. Therefore, a mechanical mechanism study of non-spherical particle migration in a microfluidic chip is essential for more precise isolation of target particles. With the manipulation of non-spherical particles, refined disease detection or medical intervention for human beings will be achievable in the future. In this review, fabrication and manipulation of non-spherical particles are discussed. Firstly, various fabrication methods for non-spherical microparticle are introduced. Then, the active and passive manipulation techniques for non-spherical particles are briefly reviewed, including straight inertial microchannels, secondary flow inertial microchannels and deterministic lateral displacement microchannels with extremely high resolution. Finally, applications of viscoelastic flow are presented which obviously increase the precision of non-spherical particle separation. Although various techniques have been employed to improve the performance of non-spherical particle manipulation, the universal mechanism behind this has not been fully discussed. The aim of this review is to provide a reference for non-spherical particle manipulation study researchers in every detail and inspire thoughts for non-spherical particle focused device design.

## 1. Introduction

With the ever-increasing popularity of microfluidic techniques, the microfluidic chip has become one of the preferred methods for label-free particle manipulation in many fields, such as biomedicine [1], drug delivery [2,3,4], food [5], agriculture [6] and environmental pollution [7]. Moreover, minimal sample and reagent consumption, simplified operation, fast detection and fairly low costs make the microfluidic chip a competitive candidate solution for disease portable point-of care testing (POCT) [8], which is significant for early diagnosis of disease and better cure rate for patients.

Conventional microfluidic research is typically based on the perfect spherical virtual model for numerical simulation or spherical polystyrene microparticles for experiments. However, in real situations, there is a tremendous variety of shapes of particles which need to be studied for precise manipulation of them. For instance, biconcave disk-like healthy human red blood cells (RBCs), sickle-like infected RBCs [9] or rod-like Escherichia coils [10] are usually sorted for disease detecting. The separation of sperm cells from white blood cells is an essential preparation procedure for assisted reproductive technologies (ART) [1]. Even though many researchers have tried to develop the movement theory of non-spherical particles in a shear flow, there are still relatively few generalizations for non-spherical particle focusing that can be employed as references for microfluidic chip design.

The typical micro scale particle manipulation methods with microfluidics can be classified into active and passive techniques [11]. The active techniques, including acoustophoresis [12], optical tweezers [13,14], di-electrophoresis(DEP) [15,16,17] and magneto-phoresis [18,19], have attracted a great deal of attention for their competitive advantages of precision and accuracy. The external force fields added in the microfluidic device are capable of providing strong discrimination for different particles, but the external force can be easily overcome by hydrodynamic flow, which limits the flow intensity and the microfluidic chip throughput. In addition, the external force field generation needs additional high-price costs for expansive equipment and space occupation, which may limit the application in portable devices. On the contrary, passive techniques are effective for efficiently focusing and separating particles with the hydrodynamic force of the relatively stronger flow intensity. Pinched flow fractionation (PFF) [20,21], deterministic lateral displacement (DLD) [22] and inertial microfluidics [23,24] are all passive methods. Especially the inertial microfluidic channels, which emerged in 2007 [25] and utilize the particle lateral migration phenomenon [26,27], are popular for their extremely high throughput, simple fabrication process and easy control. Particle inertial focusing was first reported by Segré and Silberberg in 1961. In a circular straight tube, dispersed macroscopic spherical particles gradually migrate into an annular region ~0.6 times the tube radius between channel center and wall, the well-known Segré-Silberberg annulus. Deformability, size or shape based particle sorting can be realized through small difference of their equilibrium positions [28,29].

Motion of ellipsoid or rodlike particles in an unbounded linear shear flow was first described by Jeffery’s equation [30] as

*T* = (*α*+1/*α*)×2π/*γ* (1)

where *T* is the period of rotation, *γ* is shear rat and *α* is particle aspect ratio. Tohme et al. have reviewed the non-spherical particle transportation in the straight inertial microchannel with square cross section [31]. Three modes of ellipsoid particle rotation named “kayaking”, “tumbling” and “log-rolling” were introduced. However, the influence of particle rotation on the particle lateral migration is still too complex to be completely understood. Behdani et al. [32] reviewed shape-based particle separation techniques including typical active and passive techniques and reported that most researchers of non-spherical particle separation focused on rod-like particle separation, and other shape particles have not been studied as much. In addition, there is no complete theory to describe shape effect on migration behavior.

To accelerate the process of particles focusing on their equilibrium positions, some other channel patterns were designed to bring secondary flow into the cross section. One of the typical channel patterns is the spiral microchannel, which applies Dean flow as the secondary flow to separate different shaped particles. Recently, several researchers have successfully employed spiral microchannels to separate non-spherical particles with different shapes [33,34]. New geometric parameters of the particle have been defined to predict the possibility of focusing occurrence. Furthermore, some researchers have added elastic force from viscoelastic fluid to the inertial microfluidic channel to realize high-purity separation for different shaped particles. The combination of inertial and elastic effects may be an approach to the efficient manipulation of particles with high precision.

**Figure 1:**
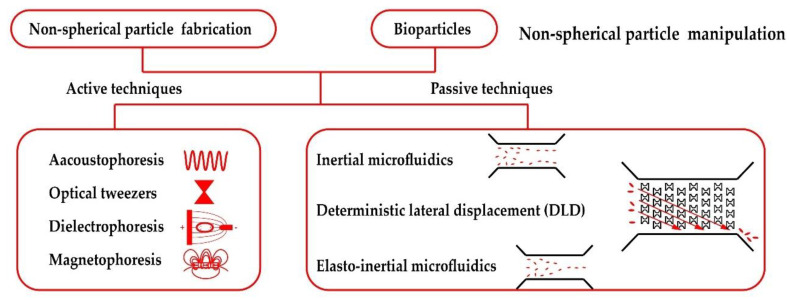
Non-spherical particle manipulation techniques.

The aim of this review is to provide references for non-spherical particle fabrication and manipulation research from a general point of view. As illustrated in Figure 1, fabrication, active and passive manipulation methods of non-spherical particle in microfluidic chip are discussed. Acoustic, optical, dielectric and magnetic methods will be introduced as active manipulation techniques. Then, inertial microfluidic channels and DLD structures are shown as passive techniques to manipulate non-spherical particles with high throughput. Subsequently, different shaped particle focusing and separation in viscoelastic fluid are discussed and compared with inertial microfluidic channels. Finally, the perspectives of future directions for non-spherical particle manipulation are given. It is hoped that this review will provide references for relevant researchers into variously shaped microparticle migration.

## 2. Non-Spherical Particle Fabrication Methods

As the sample of manipulation targets in experimental microfluidic studies, the demand for different shaped and sized particles is tremendous. Even though microscale non-spherical particle generation is challenging, there are some typical fabrication methods which are relatively mature to be considered, including spherical particle stretching, stop flow lithography, droplet microfluidics, optofluidic technique and so on.

Based on spherical particle generation, particle stretching is a direct method to prepare ellipsoidal particles [35]. Spherical polystyrene particles can be dispersed in a solution of polyvinyl alcohol to form a thin film through evaporation. Then the strips of the film are heated and stretched to convert the spherical particles into ellipsoids. This fabrication method helped Masaeli et al. [36] to study the focusing of ellipsoids with the same volume but different shapes. Except for ellipsoid particles, polystyrene pillars can be conveniently obtained through a polydimethylsiloxane (PDMS) mold with holes which are fabricated through a typical soft lithography method [37]. Conventional manufacturing methods are still worth learning for microparticle preparation of various shapes.

The basic process of stop flow lithography is that the UV-curable prepolymer solution is injected into the microfluidic channel at first, and the patterned UV light through the photo mask can define the particle shapes [38]. The obvious limitation of this method is that the synthesized particles are shaped in 2D, and the top and bottom of particle surface are flat. These 2D particles are difficult to fully simulate bioparticles which are in real world with various 3D shapes.

**Figure 2:**
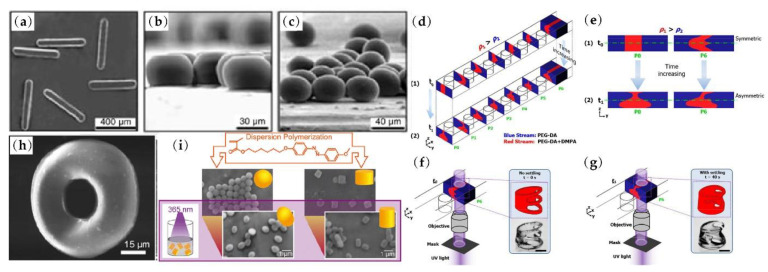
Non-spherical particle shapes for fabrication: (**a**) rods; (**b**) disks; (**c**) ellipsoids from droplet microfluidics. 4D optofluidic fabrication with (**d**) cross sections of UV-curable fluid stream passing through sequential six pillars, (**e**) cross sections of beginning and after stream shape forming with symmetric or asymmetric flows, 3D particle creation with (**f**) symmetric flow and (**g**) asymmetric flow through optofluidic fabrication. (**h**) Typical toroidal polymer particle fabricated through solidification and (**i**) different shaped non-spherical uniaxial azobenzene polymer particles under UV- or white-light irradiation. Figures (**a**–**c**) are reprinted with permission from [40], Copyright 2005 John Wiley and Sons. Figures (**d**–**g**) are reprinted with permission from [41], Copyright 2020 RSC Publishing. Figure (**h**) is reprinted with permission from [42], Copyright 2009 John Wiley and Sons. Figure (**i**) is reprinted with permission from [43], Copyright 2020 American Chemical Society.

In a microfluidic droplet generator, the liquid material of the particle is forced into a narrow orifice with sheath flow to continuously form droplets [39] and the shape of the droplets can be confined into rods, disks or ellipsoids as shown in Figure 2a–c. Mixed with a photo-initiator, monomers of droplets can be photopolymerized by illumination with ultraviolet (UV) light, and thus the droplets can be solidified into different shaped particles in situ [40]. There are many other strategies to obtain various 3D particle shapes considering the structure of the channel, the distribution of fluid and the material characteristics of particles. The optofluidic technique combines lithography and fluid inertia. Pillars in microchannel and different UV light patterns make 4D fabrication of complex structured particles 4D possible [41] (see Figure 2d–g). Through the laminar axisymmetric flow distribution, non-uniform solidification of the polymer solution droplets results in the fabrication of monodisperse toroidal polymer particles as in Figure 2h [42]. Another interesting non-spherical particle fabrication method is based on the fact that azobenzene polymer particles are light-responsive and they can change their shapes to nearly spherical, oval or short cylindrical shapes under UV- or white-light irradiation, as shown in Figure 2i [43]. The dimension of the generated particles in this research is approximately 1μm, which limits the application of non-spherical particle fabricated by this method as a substitute for a real bioparticle.

From the discussion above, it can be summarized that microparticles of arbitrary shapes are still difficult to generate. The number of achievable shapes of the fabricated particles is far from sufficient, which greatly limits the simulation of bioparticles by synthetic particle. Fabrication techniques of complex-shaped particles still need to be improved in the future to solve the shortage of research samples.

## 3. Non-Spherical Particle Active Manipulation under External Force Fields

Active techniques for non-spherical particle manipulation utilize external forces as the driving forces to manipulate the target particles. Acoustic force, optical tweezers, DEP and magnetic force are usually applied to particle manipulation with a high and precise requirement for certain particles which are sensitive in response to external force fields.

**Figure 3:**
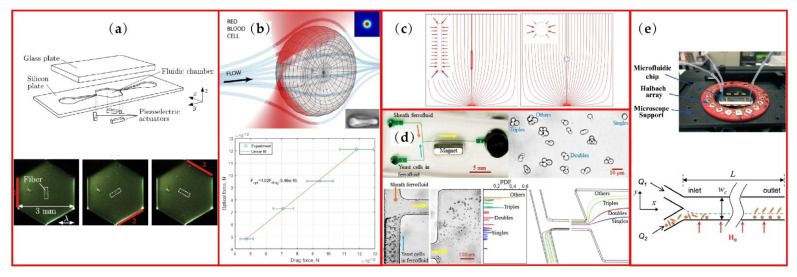
Active manipulation methods of non-spherical particles: (**a**) sketch of micro device for fiber rotation and images of fiber rotation process; (**b**) RBC trapped by single beam and the optical force estimated from the drag force; (**c**) electric field stream-line and DEP force arrows around rod and spherical particles; (**d**) T-shape microchannel under uniform magnetic field, yeast cells sample and experimental results of magnetic fractionation; (**e**) microfluidic chip in a uniform magnetic field and schematic of separation in the microchannel. Figure (**a**) is reprinted with permission from [48], Copyright 2015 Springer Nature. Figure (**b**) is reprinted with permission from [52], Copyright 2018 Springer Nature. Figure (**c**) is reprinted with permission from [37], Copyright 2015 Springer Nature. Figure (**d**) is reprinted with permission from [63], Copyright 2017 AIP publishing. Figure (**e**) is reprinted with permission from [65], Copyright 2017 RSC publishing.

Acoustic force has been a relatively mature method for particle separation. Petersson et al. separated lipid particles from erythrocytes through acoustic standing wave forces [44]. The throughput is high, but the separation purity is not satisfying, as shown in Table 1. Nam et al. and Wu et al. separated platelets and exosomes from whole blood through surface acoustic waves [45,46]. The separation purity of this method increased greatly, but the throughput declined fast. However, the separation of acoustic standing waves and surface acoustics waves was conducted mainly according to the difference of particle size, not particle shape. For non-spherical particles, research has usually focused on the acoustic radiation effects on particles. To analyze and control different shaped particle rotation, the influence of acoustic radiation torque on the rotation of fibers and other non-spherical particles was researched [47,48] (see Figure 3a). The acoustic radiation force and torque acting on different shapes were calculated for different orientation angles with a 3D numerical model [49]. Noorpoor et al. studied the effect of acoustic radiation force on the settling velocity of a vertically falling non-spherical particles in incompressible Newtonian fluid [50]. However, the analytical results show that the influence of particle sphericity on the decreasing velocity of particles was negligible.

Another nondestructive particle trapping method uses optical tweezers. With this method, particles can be sorted by size or refractive index. The sorting efficiency can approach as high as 100%, but the flow speed is as slow as 35 μm/s [13]. Most research into optical tweezers’ effects on non-spherical particles focus on particle motion and separation. Nieminen et al. calculated the force and torque on the optical trapped non-spherical particles, measuring the scattered light using electromagnetic scattering theory [51]. Bui et al. conducted the absolute calibration with a position-sensitive detector (PSD) for the paired position and force measurements of arbitrarily shaped particles in optical tweezers [52]. They used a single beam optical trap to analyze the optical force on a RBC through the comparation of the drag force as shown in Figure 3b. Zhu et al. [53] reviewed the studies of optical tweezers on the membrane deformation, electrical properties and manipulation of RBCs. The dynamic cell–cell interaction between red blood cells was also included in this review and showed the discrete cilia on the cell surface [54]. Utilizing the laser tweezers as a tool to tear the adherent RBC off from the endothelial cell, Kapkov et al. [55] carried out the measurements of the interaction forces between the individual RBC and the endothelial cell. Another interesting application of optical tweezers is to trap an ellipsoid micron particle as an optically levitated rotor which can be analog to a liquid floated gyroscope [56]. Researchers using optical tweezers on non-spherical particles mainly focus on particle rotation and cell–cell interaction. It is difficult to continuously separate particles by shape through optical force.

Di-electrophoresis is a phenomenon in which a neutral particle migrates in the fluid field driven by the polarization effects of the nonuniform electric fields [57]. Positive DEP cells move towards the strong electric field region, while negative DEP cells move to the weak electric field region. Viability of cells can be a typical indicator for DEP cell separation [15,58], which provides label-free discrimination of viable cells from nonviable ones. For particle shapes, Song et al. showed that rod-shaped particles experience larger DEP force and migrate faster than spherical particles with similar mass [37], which is a potential method for different shaped particle separating. The electric field streamline and DEP force are both illustrated in Figure 3c. DuBose et al. [59] successfully separated spherical and peanut-shaped particles through DEP force in an asymmetric double-spiral microchannel. Both shape and size of particles can be intrinsic properties simultaneously for particle separation. In their research, the typical throughput of this shape-based particle separation was estimated to be 5 μL/h owing to the weak effects of DEP (see Table 1). Efforts have been made to increase the throughput of DEP non-spherical particle focusing [60], which is combined with microchannel with grooves, but shape-based particle separation by DEP is still difficult to efficiently accomplish.

Magnetic technique can be another label-free method to conduct shape-based particle separation. Through biocompatible ferrofluids, Kose et al. realized the isolation of live RBCs from sickle cells and bacteria [61]. Inspired by this idea, Zhou and Xuan from Clemson University separated equal-volumed spherical and peanut-shaped particles through dilute ferrofluids [62] and their group successfully applied this technique to yeast fractionation as shown in Figure 3d [63]. Yeast cells were separated into four groups: singles, doubles, triples and others. Simulation results of COMSOL^®^ 5.1 reasonably agreed with the trajectories of the yeast cells in the experiment. However, the throughput of this method was not obviously higher than that of the DEP method as shown in Table 1. Matsunaga et al. focused on and sorted the magnetic ellipsoidal particles in a straight microchannel [64]. Utilizing a static uniform magnetic field, they pinned the orientation of the magnetic particle during migration and focused the ellipsoidal particles to arbitrary transverse target positions through magnetic field control. Zhou et al. [65] from Missouri University of Science and Technology achieved shape-based separation in a uniform magnetic field, as shown in Figure 3e. The shape-dependent lateral migration can be ascribed to asymmetric rotation of particles and the degree of rotational asymmetry can be directly affected by the direction and strength of the magnetic fields [66]. What is interesting is that this method uses shape-dependent magnetic torque, not magnetic force. Cao et al. [67] numerically investigated the influence of inlet flow velocity, magnetic field direction and particle shape on the lateral migration of elliptical particles. Lower inlet flow velocity and appropriate magnetic field can lead particles to the equilibrium position more quickly with a non-rotational direction and elliptical particles with different aspect ratios can be separated. The influence of magnetic field intensity on the speed of particle lateral migration was further studied by Zhang et al.’s numerical model [68] and the difference between paramagnetic particle and ferromagnetic particle lateral migration under the same flow and magnetic conditions was also discussed [69]. In particular, blood cells separation is naturally appropriate for magnetic method because of different response characteristics of white and red blood cells to the magnetic field and many researchers have verified the feasibility of blood cells separation via magnetic field [70,71].

From the above discussion, there are not many researchers reporting on non-spherical particle continuous separation via acoustic technique or optical tweezers and most relevant research was conducted in the static fluid field to study the rotation and mechanical characteristics of non-spherical particles. For DEP and magnetic techniques, the typical Reynolds number (*Re*) is low and usually <1 [59,65], which means that, even though the discrimination capacity of these external fields is strong so that viability, shape or magnetic property of particles can be their separation indicators, throughput is still the biggest limitation for dielectric and magnetic separation.

**Table 1:** Recent advances in active manipulation of non-spherical particles under external force fields.

Targets	Application	External Force Fields (Sample Flow Rate)	Performance	Reference
Erythrocytes/ Lipid particles	Separation of erythrocytes and lipid particles	Acoustic standing wave forces/ (0.3 mL/min)	Separation purity erythrocytes:>70% lipid particles:>80%	[44]
Platelets	Separation of platelets from whole blood	Standing surface acoustic waves (0.25 μL/min)	Separation purity Platelets: nearly 98%	[45]
Exosomes	Separation of exosomes from undiluted Blood	Standing surface acoustic waves (4 μL/min)	blood cell removal of rate:>99.999%.	[46]
Spherical particles/ peanut-shaped particles	Separation of spherical particles and peanut-shaped particles	C-iDEP (5 μL/h)	Separation purity spherical particles:>80% peanut-shaped particles:>80%	[59]
Sickle cells	Separation of sickle cells from healthy red blood cells	Ferrofluids (-)	Separation efficiency:75.2% Separation purity:89.3%	[61]
Yeast cells	Separation of yeast cells of different morphologies	Ferrofluids (Slightly smaller than 9 μL/h)	Optimal separation of yeast cells of different morphologies	[63]
Ellipsoidal Magnetic Particles	Separation of Ellipsoidal Magnetic Particles in Microchannels	Static magnetic field (-)	Controlling elliptical magnetic particles to any lateral position by a static magnetic field	[64]
Ellipsoidal Magnetic Particles	Separation of ellipsoidal and spherical l magnetic particles	Uniform magnetic field (0.2 µL/min)	Complete separation with shape-dependent lateral migration of the particles	[65]

## 4. Non-Spherical Particle Passive Manipulation in Microchannels

### 4.1. Inertical Focusing in Straight Microchannels

**Figure 4:**
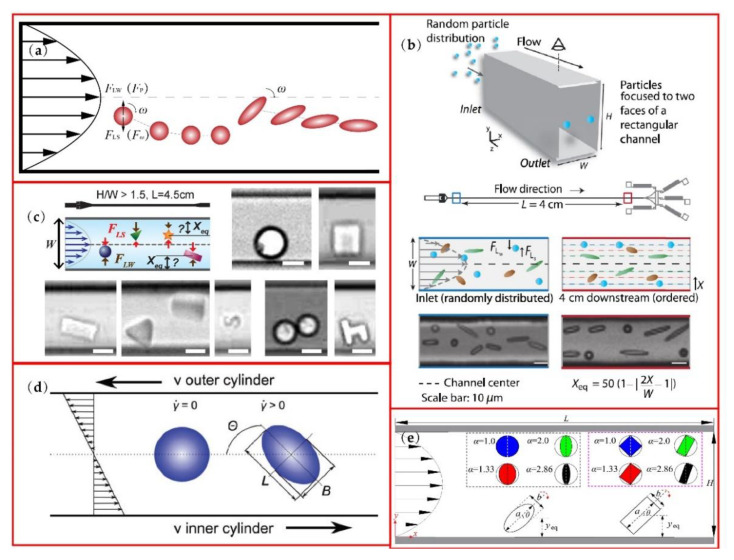
Non-spherical particle manipulation in straight microchannels: (**a**) sketch of particle focusing in a Poiseuille flow; (**b**) ellipsoids with different aspect ratios focusing, reprinted from [36]; (**c**) microparticles with various shapes in a straight microchannel; (**d**) non-spherical capsule rotation in a shear flow; (**e**) elliptical particle and rectangular particle migrating in a channel flow. Figure (**c**) is reprinted with permission from [29], Copyright 2011 AIP publishing. Figure (**d**) is reprinted with permission from [75], Copyright 2015 Elsevier. Figure (**e**) is reprinted with permission from [84], Copyright 2021 Elsevier.

Inertial microfluidics is capable of focusing particles at their equilibrium positions with extremely high throughput utilizing the hydrodynamics of fluid and the typical *Re* of inertial microfluidics ranges from ~1 to ~100 [72]. Generally, the inertial focusing of particles can be ascribed to the balance of shear gradient induced force *F*_LS_ directed towards the walls and the wall induced lift force *F*_LW_ directed towards the center, as shown in Figure 4a. Another theory to explain particle focusing is that it is under competition from particle rotation induced force *F*_ω_ and the pressure induced by force *F*_p_ on the particle, which focuses on the interaction between particle and the fluid around it [73]. Micro scale particle focuses into the Segré-Silberberg annulus in a confined round tube. For square and rectangular channels, the equilibrium positions of particles change because of the different velocity distribution. In a square channel, particles focus into four equilibrium positions near the middle of the walls, while in a rectangular channel, four equilibrium positions reduce to two positions close to the middle of the longer walls where velocity gradient is larger, as shown in Figure 4b.

For non-spherical particle separation, rotation is a very important and complex influence factor on particle focusing. Many researchers have tried to describe the mechanism of non-spherical particle lateral migration, including migration mode, dimension parameters, shape character and so on. Hur et al. [29] investigated the inertial focusing of particles with various shapes in straight microchannels and defined the rotational motion of cylinders and disks during inertial focusing as “tumbling” and “log-rolling”, respectively. Based on the experimental research, rotational diameter *D*_R_ was found as the important dimension parameter for lateral equilibrium position *X*_eq_ of non-spherical particles. Except for “h-shape” particles, all tested particles, as shown in Figure 4c, had a closer equilibrium position to the channel center with increasing *D*_R_. The high asymmetry of the h-particle may lead to different behavior from that of other particles. To further study the influence of dimension parameters on lateral migration of non-spherical particle, Su’s group defined another two diameters of a cylindrical particle besides *D*_R_ [74]. In their numerical simulation research, *D*_A_ was defined as the axial length of the cylindrical particle and the equivalent diameter of a cylindrical particle is the diameter of the corresponding spherical particle that has the same equilibrium position. With the Re increasing, equivalent diameters of cylindrical particles also increase. Specifically, when *Re* = 50, equivalent diameter can be chosen as *D*_A_ and, as the *Re* increases to 200, equivalent diameter gradually turns to *D*_R_.

In the research of Masaeli et al., with similar volume, the larger aspect ratio α particle has longer *D*_R_ and closer lateral equilibrium position to the channel center (see Figure 4b) [36]. This may be explained by the fact that particles with larger aspect ratio α rotate slowly according to Jeffery’s theory, as in Equation (1). The attenuated rotation and the relative dominance of *F*_LW_ or *F*_p_ lead to a closer equilibrium position to channel centerline for larger α ellipsoids. In addition, because of the confinement of the walls, the larger aspect ratio α particle will be pushed away from the wall when the major axis rotates to an orientation perpendicular to the wall, which may attenuate the particle rotation further and focus particles close to the channel center. Unverfehrt et al. found that, in a simple shear flow, there are two basic oscillation phenomena of non-spherical capsules which can be observed in the experiments [75]. A tumbling mode describes the periodic variation of the inclination angle *θ* (see Figure 4d) between +90° to −90° during continuous rotation of capsules. With shear rate increasing, capsules enter the swinging mode in which inclination angle oscillates around a positive value. Meanwhile, the swinging mode is usually accompanied by the tank-treading motion if the capsule is deformable and surrounded by a membrane, and the reason for this phenomenon might be that elastic energy of the membrane and the shape memory effect this.

The Lattice Boltzmann method (LBM), as a mesoscopic scale numerical simulation, has been a useful tool to study the interaction between the particle and the flow around it [76,77,78]. The physical meaning of this method is clear, and it is naturally appropriate for particle simulation and convenient for building up complex boundaries [79,80]. Moreover, computation time can be less, because of the easy parallelization of the code. Dissipative particle dynamics (DPD) is another mesoscale fluid field computation method, but mesh-free, which is also approprate to be employed in particle migration research [81]. The numerical calculation method has been a more and more popular tool for particle and fluid interaction study.

Ladd [82,83] first applied LBM in the simulation of particle migration. Recently, several researchers have employed the mesoscopic simulation methods to investigate performance of different shaped particles in lateral migration. Huang et al. analyzed inertial migration of neutrally buoyant prolate and oblate particles in a Poiseuille flow using DPD. Hu et al. [84] calculated the migration of elliptical particles and rectangular particles in a power-law flow (see Figure 4e—a and b are long and short axes for elliptical particle and length and width for a rectangular particle and H is channel height) with LBM. They both found that the equilibrium position of lateral migration is mainly under the influence of aspect ratio α (α = a/b), blockage ratio k (k = a/H for elliptical particle, k = (a^2^+b^2^)^0.5^/H for rectangular particle) and Reynolds number. With higher blockage ratio k and higher Reynolds number, the particle equilibrates more closely to the channel centerline. Huang et al. conformed that, with the same volume, the equilibrium position of the higher aspect ratio α particle is closer to the channel center as the results in Masaeli’s research [36]. Prolate and oblate particles migrate to the equilibrium positions closer to the center than in a sphere of the same volume. Hu et al. also explored the higher power-law index n leading the particles closer to the channel center. Different from the research above, they compared the migration of particles with similar D_R_ and found that lower aspect ratio α led the particles to equilibrate closer to the channel center. In addition, for oblate spheroid particles, the LBM calculation results of Nizkaya [85] show that the equilibrium position of an oblate spheroid particle depend only on its equatorial radius (R_eq_) rather than polar radius (R_po_) during their “log-rolling” motion. Lashgari et al. [86] numerically studied the inertial migration of sphericle and oblate particles in straight square and rectangular channels using the immersed boundary method to calculate the interaction between particles and surrounding flows. In a square channel, oblate particles finnaly equilibrate at the centers of the walls like spherical particles, but migrate with tumbling motion and longer downstream focusing length and rotate remaining vertical to the adjacent walls, no matter the initial position and orientation of the particles. In the rectangular channel, oblate particles are also likely to focus at the center of the longer walls.

### 4.2. Inertial Focusing in Secondary Flow Microchannels

**Figure 5:**
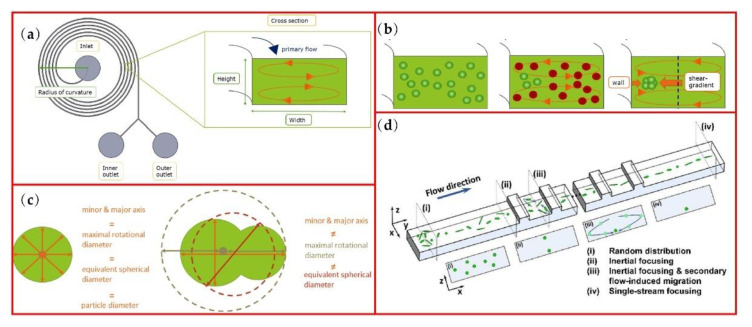
Non-spherical particles focusing in spiral microchannels: (**a**) Dean flow in the cross section of a spiral microchannel; (**b**) focusing of unstable particles (red) to stable positions (green); (**c**) dimension parameters for spherical and non-spherical particles. Another secondary microchannel of (**d**) ellipsoid-shaped E. gracilis focusing with different aspect ratios in a stepped microchannel. Figures (**a**–**c**) are reprinted with permission from [33], Copyright 2018 AIP publishing. Figure (**d**) is reprinted from [87], Copyright 2016 RSC publishing.

The application of secondary flow can effectively accelerate the lateral migration of particles. In spiral microchannels, the Dean flow, as a secondary flow in the cross section of the channel, is able to separate microparticles more efficiently according to their sizes. As shown in Figure 5a [33], Dean flow consists of two counter-rotating vortices in the cross section and Dean force can be balanced with *F*_LW_ and *F*_LS_ to focus the particles near the inner wall of the spiral microchannel (see Figure 5b). Utilizing this phenomenon, Roth et al. realized non-spherical particle focusing and defined several different diameter parameters of non-spherical particles for particle focusing study, including minor axis (*min.a*) and major axis (*maj.a*), the equivalent spherical diameter (*esd*) and the maximal rotational diameter (*mrd*), as shown in Figure 5c. These parameters are equivalent for spherical particles but non-equivalent for non-spherical particles. This study found that, when the ratio of the equivalent spherical diameter of the non-spherical particles to the hydraulic diameter (*D_h_*) of the channel is greater than 0.07 or the ratio of the maximum rotational diameter (or major axis) to the hydraulic diameter is greater than 0.1, the non-spherical particles are focused. Except for minor axis, all other parameters can be used to predict successful focusing.

The spiral microchannel is a relatively mature structure for particle separation. For instance, this kind of channel can be applied for separating sperm cells from white blood cells as a protocol of intrauterine insemination sperm preparation for assisted reproductive technologies [1,34]. The average recovery of this instrument is 86% with 5 mins of operating time, which can efficiently reduce the volume of the sperm sample to the clinically required level.

Another structure for the focusing of different shaped particles is the stepped microchannel as shown in Figure 5d [87]. The combination of inertial focusing and secondary flow in the cross section is capable of focusing ellipsoid particles with different aspect ratios at the single equilibrium position. In detail, inertial flow firstly focuses the different shaped particles at the center of a low aspect ratio channel with two equilibrium positions, and then the secondary flow caused by the stepped structure can reduce the two equilibrium positions to a single one. 3D focusing can be easily achieved through this channel pattern.

### 4.3. Non-Spherical Particle Separation in DLD Microchips

**Figure 6:**
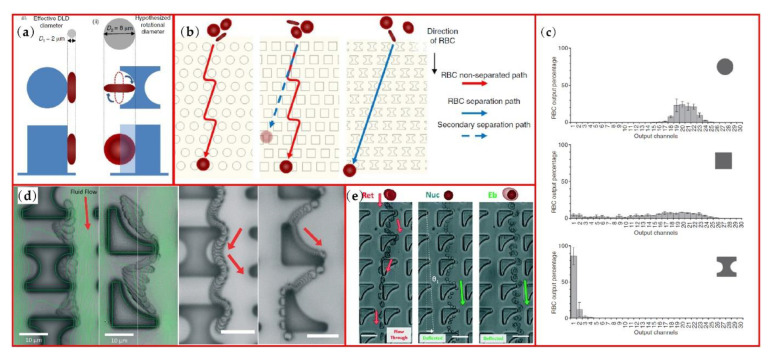
Non-spherical particles focusing in DLD microchannels: (**a**) difference of particle rotation in round and I-shaped pillars DLD; (**b**) projected paths of RBCs passing through round, square and I-shaped pillars; (**c**) results of the RBC separation for round, square and I-shaped pillars; (**d**) RBCs and spherical beads separation movement tracking in I-shaped and L-shaped pillars; (**e**) reticulocyte separation from expelled nuclei and nucleated erythroblasts in inverse-L pillar arrays. Figures (**a**–**c**) are reprinted with permission from [88], Copyright 2013 Springer Nature). Figure (**d**) is reprinted with permission from [89], Copyright 2014 RSC Publishing. Figure (**e**) reprinted with permission from [90], Copyright 2020 RSC Publishing.

DLD microchannel is constructed from pillar arrays and known as a high-resolution microfluidic device. Conventional pillars are round-shaped, and each row of pillars is shifted horizontally. Different sized particles can be injected at the same position and separated with a high resolution of ~10 nanometers [22]. In the experimental research of Zeming et al., I-shaped pillar array was more effective than square or round pillar array for RBC separating because of the non-spherical particle rotation induction of the I shape [88]. As shown in Figure 6a, I-shaped pillar has two protrusions to induce the disc-shaped particle rotation and a semicircular groove to accommodate the rotation.

Thus, RBCs can be separated from other blood cells through the DLD structure with I-shaped pillars. The trajectory of RBCs is an oblique line to the left side output (see Figure 6b). The results of their output graph confirm the higher efficiency of I-shaped pillars compared to round and square pillar DLDs (see Figure 6c). Different pillar shapes including Anvil, T-shape and L-shape were also investigated [89]. Protrusions and grooves of pillars can induce or confine the rotation of a particle and the combination of protrusions and grooves can change the orientation of non-spherical particles. Based on their experimental results, I-shaped pillars can separate both spherical and non-spherical particles, but L-shaped pillars can only separate non-spherical particles (see Figure 6d). These special shaped pillars can be expected to be applied for spherical particles, blood cells and rod-shaped bacteria separation.

The comparison of spiral microchannel and DLD microchannel with inverse-L pillars was conducted through bioprocessing, separating human reticulocytes from erythroid cultures which contain mainly reticulocytes, nucleated erythroblasts and expelled nuclei [90]. The reticulocytes are the preferable invasion targets for malaria parasites and have a very small percentage of 0.5–2.5% in whole blood cells, which provides a good reason to enrich the reticulocytes with microfluidics. Utilizing the deformability of reticulocytes, reticulocytes migrate with the surrounding flow and can be separated from nucleated erythroblasts and expelled nuclei, as shown Figure 6e.

Non-sphericity is one of the characteristics of cells. Most of the cells in the organism and bacteria in nature are non-spherical. Therefore, exploring the movement mechanism of non-spherical particles in the microfluidic channel is of great significance for cell focusing and sorting and also conducive to accurate diagnosis and precision medicine in the future. However, the motion mechanism of non-spherical particles is still in the exploratory stage. Table 2 summarizes the research on the motion mechanism of non-spherical particles in microchannels.

### 4.4. Comparison among Active and Passive Non-Spherical Particle Manipulation Methods

Compared with the active non-spherical method, passive non-spherical particle manipulation has become mainstream in recent years owing to the usually tens or hundreds of times higher throughput and more reasonable cost with no external force fields. There is no significantly better performance so far for particle separation active methods, just based on shape difference. Therefore, relatively fewer researchers focus on active non-spherical particle manipulation. However, active methods are still irreplaceable for their high sensitivity to the distinctness of particles, such as viability, ferromagnetic and immunological characteristics.

Among passive non-spherical particle manipulation microchannels, the theory of non-spherical particle focusing is usually studied in a straight microchannel because of the simplicity of the fluid field, but the equilibrium positions of different sized particles are relatively close and the time consumption is relatively longer. The addition of second flow accelerates the focusing process greatly and realizes the single equilibrium position with high volumetric throughput. DLD microchannel is a competitive manipulation method for non-spherical particles which accomplishes extremely high-purity separation and high resolution, even though the fabrication of the DLD microchannel is a little more difficult relatively and the throughput is significantly lower compared with that of other inertial microchannels, because of the larger flow resistance of pillars as shown in Table 1.

## 5. Non-Spherical Particle Manipulation in Non-Newtonian Fluid

**Figure 7:**
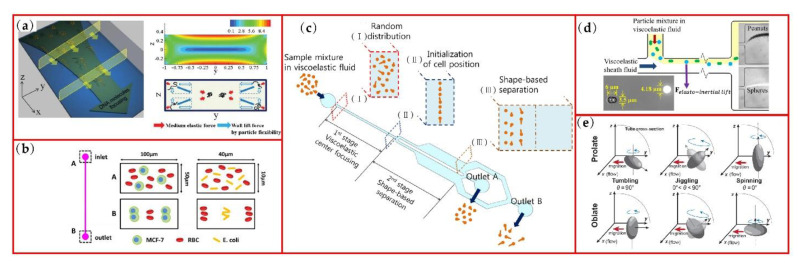
Non-spherical particle manipulation under viscoelastic flows: (**a**) 3D DNA focusing under viscoelastic flows; (**b**) rectangular cross section illustration of separation of MCF-7 cells, RBCs and E.coils; (**c**) schematic of shape-based separation of candida cells in a viscoelastic fluid, reprinted from [95]; (**d**) separation of spherical and peanut-shaped particles under viscoelastic flows; (**e**) possible modes of motion for spherical particles under viscoelastic flows in a circular tube. Figure (**a**) is reprinted with permission from [93], Copyright 2012 RSC Publishing. Figure (**b**) is reprinted with permission from [94], Copyright 2015 American Chemical Society. Figure (**d**) is reprinted with permission from [21], Copyright 2015 American Chemical Society. Figure (**e**) is reprinted with permission from [99], Copyright 2020 John Wiley and Sons.

Elasto-inertial focusing is a relatively new microparticle manipulation method which overcomes the low throughput of external force fields and reduced sensitivity for various shapes of non-particles in an inertial flow. In viscoelastic flow, there is an additional elastic force exerting on particles. The combination of inertial and elastic force will focus particles to the channel centerline, which provides convenience for particle detection [91,92]. The applications of the elasto-inertial focusing have been expanded to flexible DNA molecules focusing [93], MCF-7, RBC and *E. coli* separation [94] and shape-based two-stage candida cell separation [95] as shown in Figure 7a–c. Compared with Newton fluid, viscoelastic flow is more sensitive to the shapes of non-spherical particles. As shown in Figure 7d, spherical and peanut-shaped particles with similar volume can be separated in the viscoelastic flow [21] and this structure was modified to a sheath-free microchannel in the later research [96]. Yuan et al. [97] separated the cyanobacterial anabaena by shape using a combination of inertial flow, viscoelastic flow and secondary flow in contraction-expansion microchannel, and this integrated system may be the future of non-spherical particle precise manipulation. Compared with Newton fluid, viscoelastic flow is more sensitive to the shapes of non-spherical particles. Table 3 summarizes the sorting performance of non-spherical particles under viscoelastic fluids.

Recently, the migration mechanism behind various motion modes of non-spherical particles has been preliminary studied through numerical simulations. D’Avino et al. [98] utilized 3D numerical simulation to investigate the translational and orientational dynamics of a spheroid particle in a viscoelastic wide slit microchannel flow. Whether the particle migrates towards the channel centerplane or the wall can be decided according to the initial distance of the particle from the centerplane and the initial particle orientation. Tai et al. [99] numerically studied the migration of non-spherical particles in second-order viscoelastic fluid flows and found that jiggling is the most likely motion for both prolate and oblate particles, since tumbling and spinning mode require specific orientation as shown in Figure 7e. However, regardless which mode particles experience, they migrate to the channel centerline. The different flow profiles may alter the orientation dynamics of non-spherical particles, but their influence on the particle migration speed towards the centerline is not obvious. In a power-law fluid, the equilibrium positions of elliptical and rectangular particles are related to the power-law index *n*, which means that particles in shear-thickening fluid laterally migrate closer to the channel centerline than in the shear-thinning fluid [84].

Furthermore, considering the confinement effects of the wall, Kawaguchi et al. studied the influence of particle-wall distance and rotation of round and elliptical particles on effective viscosity by results calculated from LBM [100]. They found that the effects of particle shape on effective viscosity can be enhanced when particles flow near the channel walls. All the simulation research above proves also that the numerical simulation method is a powerful method to investigate motion modes and migration principle of various shaped particles. Details of particle motion and interactions between particles and surrounding flow can be clearly observed in the calculated data. More inspirational numerical research is emerging for non-spherical particle manipulation study.

## 6. Conclusions and Perspectives

After about three decades of development, microfluidics has become a relatively mature technique for particle manipulation. For the future of microfluidics, high precision, high efficiency and product differentiation are the development directions. Particle manipulation research was mainly based on the size difference of particles in the past, but particle shape is another important physical characteristic, and may be more valuable for bioparticle detection. In some situations, healthy cells attacked by a disease may change their shapes greatly. Like sickle cells produced by sickle cell anemia, the sickling red blood cell becomes longer and thinner. Thus, size is no longer enough to describe the different cells and shape is of great value for separation of abnormal cells for diagnosis and treatment. In this review, fabrication and manipulation methods of non-spherical particles are discussed, including non-spherical particle fabrication, active and passive techniques for non-spherical particle manipulation and non-spherical particle focusing or separation in viscoelastic flow. General information about non-spherical particle manipulation has been provided from relevant research.

For fabrication of non-spherical particles, simple shaped particles such as rods, disks, ellipsoids and toroidal particles can be obtained through droplet microfluidics. However, few of them have appeared in the commercial market so far. Stop flow lithography is capable of providing 2D arbitrary shaped particles and incapable of fabricating 3D arbitrary shaped particles. In future, 3D arbitrary shaped particle fabrication methods are expected to emerge. It is also expected that arbitrary shaped particles for custom tailoring can be provided in the commercial market.

For the non-spherical particle manipulation methods usually applied in recent years, the precision of active manipulation techniques is usually satisfactory and the active techniques are suitable for fine control of particle motion [101]. However, few researchers can be referenced for continuous separation of non-spherical particles through acoustic force or optical tweezers, and these external forces are usually employed for particle rotation and mechanical characteristic research. DEP and magnetic force are more common in continuous non-spherical particle separation with high precision, but the throughput may be sacrificed. Inertial microfluidics, as the most popular kind of passive microfluidic techniques, is known for its extremely high throughput and low costs. With the help of Dean flow or other secondary flows, efficiency can be further improved. However, the precision for non-spherical particle inertial separation perhaps cannot satisfy high requirements. DLD structure or viscoelastic fluid may overcome this dilemma. Multi-stage separator structure for DLD or elastic force from viscoelastic fluid has been experimentally validated to be effective for precision promotion while the throughput is kept at a reasonable level.

Research into the mechanism of non-spherical particle manipulation is usually conducted as cylinders, prolate or oblate particles focusing in straight microchannels. The particle shapes and the flow fields are relatively simple, which simplifies mechanism discussion. However, more complex situations should be explored in the future. Numerical simulation is becoming increasingly popular for mechanism study, especially the LBM model which has been frequently applied in recent years. Numerical simulation is appropriate for particle migration mode study since every detail of particle migration can be observed through calculation and more information about the interaction between particle and fluid can be provided by calculation, compared with the experimental research. LBM is a mesoscopic fluid field calculation method and has many advantages including clear physical understanding, easily parallelization, suitable for complex boundaries and shorter time. Therefore, various particle shapes can be easily built to fit bioparticles, which make LBM a good candidate for arbitrary shaped particle modeling in a complex microchannel in the future. Furthermore, the study of viscoelastic fluid is relatively rare now, but elastic force may be a powerful tool to precisely manipulate non-spherical particles. To overcome the relatively lower throughput of viscoelastic focusing, combination effects of viscoelastic fluid and secondary flow on non-spherical particles may be a challenging and promising field for future experimental and numerical research.

With the development of microfluidic techniques and numerical simulation methods, it can be envisioned that shape will be a more useful characteristic for particle separation, and particle identification will be refined to obtain more precise results.

## Figures and Tables

**Table 2:** Recent advances in passive manipulation of non-spherical particles in microchannels.

Particle	Microchannel	Parameter	Sample Flow Rate or Re	Performance	Reference
Non-spherical particles (except H-shaped)	Straight microchannel	*D_R_*	*Re* = 200	The equilibrium positions of the measured particle·s are all close to the center of the channel with the increase of *D_R_*.	[29]
Cylindrical particle	Straight microchannel	*D_R_,D_A_*	-	Equilibrium position for spherical particles of equal diameter with equivalent diameter, and as Re increases, the *D_A_* also increases	[74]
Ellipsoid particle	Straight microchannel	α	60 µL/min	The volume is the same, the larger the α, the closer the lateral position of the particle is to the center of the channel	[36]
*E. gracilis*	Stepped microchannel	-	100, 200 and 300 µL/min	Ellipsoidal particles with different aspect ratios focus to a single equilibrium position	[87]
Non-spherical capsules	Straight microchannel	*θ*	-	Capsules periodically change at −90° and +90° with the tilt angle *θ* during rotation (shear flow)	[75]
Ellipsoid particle	Straight Microchannels in (Power-Law Fluids)	*k, Re, n*	*Re* = 32	As *k*, *Re*, and *n* increase, the particle lateral position is closer to the channel centerline	[84]
Rectangular particles					
Oblate spherical particles	Straight microchannel	*R_eq_, R_po_*	*Re* ≤ 44	The equilibrium position of particle in a "log-rolling" motion depends only on its *R_eq_*, not its *R_po_*	[85]
Oblate particles	Square straight microchannel	*-*	*Re* = 100	Equilibrate at the center of the wall like spherical particles	[86]
	Rectangular straight microchannel			May be concentrated in the center of longer walls	
Non-spherical particles	Spiral microchannel	*esd, mrd, maj.a, D_h_*	75 µL/min	non-spherical particles focusing when *esd*/*D_h_* > 0.07 or *mrd* (or *maj.a*)/*D_h_* > 0.1	[33]
Non-spherical particles	DLD	*-*	0.1–0.2 µL/min	The protrusions and grooves of the pillars can induce or constrain the rotation of particles, and the combination of protrusions and grooves can change the orientation of non-spherical particles	[89]

**Table 3:** Recent advances in non-spherical particles in elastic-inertial microfluidics.

Targets	Application	Sample Flow Rate or *Re*	Performance	Reference
λ-DNA (*R_g_* = 0.69 μm) /T4-DNA(*R_g_* = 1.5 μm) (*R_g_*, radius of gyration)	Three-dimensional single-stream DNA molecules focusing	*Re* = 6.8 × 10^−3^	T4-DNA of 1.5 μm *R_g_* molecules are more concentrated along the channel centerline	[93]
MCF-7 cells/ RBCs	Separation of MCF-7 cells and RBCs	3 mL/h	Separation efficiency MCF-7 cells:91.4% RBCs:91.7%	[94]
*E. coli*/RBC	Separation of E. coli and RBC	0.1 mL/h	Separation efficiency E. coli:99.9% RBCs:94.1%	[94]
Spherical particles/Peanuts	Separation of spherical particles and peanuts	5 μL/h	Separation purity spherical particles:>90% Peanuts:>90%	[21]
Cyanobacterial anabaena	Separation different shapes of cyanobacteria anabaena	40 μL/min	Filament length of 5~100 μm migrated to the cavity side; 100~400 μm migrated to the channel center; 400~1000 μm migrated to the opposite cavity side	[97]
